# Cundurango

**Published:** 1871-12

**Authors:** E. Andrews

**Affiliations:** Professor of Surgery in Chicago Medical College; No. 6 Sixteenth St., Chicago


### CUNDURANGO.

By E. ANDREWS, Professor of Surgery in Chicago Medical College.

There seems to be, on the part of some of the profession, a foolish disposition to speak of this article in a spirit of partizan spitefulness, instead of a cool scientific candor. It is to be remembered that in this matter we have nothing to do with the question of whether Dr. Bliss is a rascal or a philosopher. We should only enquire what are the effects of the medicine. It will be acceptable to many, doubtless, to read what is the present state of our information on the subject.

Cundurango is a woody vine growing in the forests of the Andes, in the same region with the cinchona-tree. The singular name is a combination ot the native word *cundur*, a condor, and *ango*, a vine, and signifies condor-vine. The botanists call it *Equatoria garciana*, the first word being derived from the name of the country (Ecuador), and the second given in honor of its president, Don Garcia Morena. It is a slender, woody vine, from one to three inches in diameter, winding spirally, like our native bittersweet, and climbing to the tops of the tallest trees. The fresh stems exude a milky juice when broken, the leaves are cordate and about five inches long, the flowers are in clusters like the silk-weed, and the fruit is a large pod. In many respects the vine claims affinity with the genus Asclepias. The bark is the portion used for medicine, and the fruit is reputed among the natives to be a poison.

The facts, real or asserted, about its medicinal efficacy, are as follows:

The native tradition: An Indian in Ecuador, afflicted with some cancer or ulcer, was believed by his friends to be past help. His wife, therefore, determined to shorten his sufferings by poisoning him. Accordingly she prepared a decoction of cundurango and mixed it with his food. As he did not die, she increased the dose, but still he felt no injury; on the contrary, his ulcer, whatever it was, completely healed.

Fifty years later, Dr. Eguiguren, brother to the governor of the province of Loja, determined to test the truth of the tradition, and tried the cundurango with success on several cases of syphilis and cancer. The Doctor about this time went into politics, and paid no more attention to the matter; but his brother, the governor, tried it on a negro servant, who had a bad disease, suspected to be cancer, and cured him. The information was sent to Quito and other places, where Dr. Casares and Dr. Chiribago experimented with it. These four gentlemen report nine cases of cancer cured.

So far the statements are all on one side. On the other hand, the old bishop of that province, who remembered the Indian and his wife very well, asserted that he died very soon after he was reported cured. It is stated also that the same unfortunate result followed in the case of the negro servant of Governor Eguiguren. In short, a traveler now in Ecuador writes that every case of reported cure of cancer, which he has been able to track up, died soon after the cure was effected. It is stated in the newspapers, also, that a Dr. Myers, of San Francisco, Cal., was employed for some time in a hospital in South America where cundurango was tried in about twenty cases, and that it was a total failure in every instance.

Ecuador is too far away to admit of our sifting these contradictory statements at once, so as to evolve the exact truth. I have written to Dr. Eguiguren, Dr. Casares, Dr. Chiribaga, and several others, but many weeks will elapse before an answer can be obtained. At present we are obliged to content ourselves with the fact that the remedy has attracted the attention of surgeons there, and that some of them at least report favorably upon it. So much for the South American portion of the investigation.

During the past summer, E. R. Wing, of the U. S. Legation in Ecuador, sent about fifty pounds of the article to the Department of State at Washington, with a letter on its virtues, which was republished in about every newspaper in the United States. The cundurango sent was all distributed for experiment among Eastern surgeons, so that I was unable to get any for trial here. Of those receiving it, Dr. Bliss, of Washington, reported three cases greatly benefited, one of which is the much quoted instance of Mrs. Matthews, the mother of Vice-President Colfax. At the same time a quantity of cundurango was sent to the British government and distributed to two of the London Hospitals. From one of the institutions there is no response as yet, but the other, the Middlesex Hospital, reports that the article proved inert in their hands.—*British Medical Journal.*

The newspapers all over the United States published Mr. Wing's letter to the State Department, as well as Dr. Bliss's report of his three cases, and Vice-President Colfax's letter, stating the improvement in his mother's condition, so that cundurango got the most tremendous gratuitous advertisement ever heard of in this country.

Dr. Bliss saw that there was a prodigious speculation in store for the lucky man who should first get cundurango for sale. He therefore founded an importing house in New York, and sent his partner, Dr. Keene, at once to Ecuador, to get the first stock.

There is a mystery about the other surgeons to whom specimens were given for trial. I cannot ascertain whether they reported, or what results they observed. The statement is made that their results were unfavorable to the drug, but that some official in Washington, interested in the speculation, suppressed the reports, and, on being applied to, refused to allow them to be copied or printed. I have not been able to ascertain whether this is true or false. The first fifty pounds were thus used up, and we have only one man's report of the result, and he interested heavily in the speculation of importing the article. Still, there is no doubt that Mrs. Matthews made a surprising improvement while taking the medicine.

In August or the early part of September, Dr. Keene returned with a stock of cundurango, and began to sell at $160 a pound. The price then fell to $38, which it still holds.

Contradictory accounts come from various quarters as to results. In Chicago, one case was remarkable enough to get attention in the newspapers. This was a lady, of good circumstances and character, who had a large and very soft cancer in the breast. In January, 1870, it was so soft as to give the sense of fluctuation to the finger. In May or June of the same year I introduced an exploring needle in several places, hoping to find an abscess or a cyst, but only obtained a little blood. A severe inflammation followed the punctures, which was subdued by active treatment.

Subsequently the family physician, Dr. Hatch, renewed the exploration with a lancet. As before, there came out nothing but a little blood, but a new and severe inflammation followed, with the remarkable result of a complete sloughing out of the tumor, and an apparent progress towards healing, so that the hopes of the friends were greatly excited. The tumor, however, returned again. In August or September last the patient went to Washington, and began to take cundurango under the care of Dr. Bliss. At this time a new inflammation took place, the tumor bulged out, and on being operated on was found to be again sloughing away. The cavity began to heal, and at present is entirely cicatrized, while the patient though still suffering slight pains, feels greatly relieved, and very hopeful of the future. She thinks her sense of vigor and general health is perceptibly greater than after the first sloughing.

Prof. Byford reports that he has used cundurango in three cases of cancer with no effect whatever, except to disorder the stomach.

I have given it in six cases, but only two of them remained in the city long enough to allow me to watch the effects. One of them had a cancer taken out of the breast, five years ago, by a "plaster" applied by a quack. The tumor returned, as usual in such treatment. On November 13, I placed her upon the use of the new remedy. On the sixth day after, the lancinating pains began to abate, and she continued to feel better until the sixteenth day from commencing the remedy. She then felt worse for four days. On the twentieth day she began to improve again, and the twenty-ninth day (time of the present writing) she was still better. The scirrhus is not very much changed, but I think the red, knobby prominences are a little paler, and a little flatter. On the whole, she may be said to be somewhat improved.

Another case was one of well marked scirrhus surrounding the rectum. There was a good deal of pain, and an offensive discharge. Ordered to commence the cundurango. The third day the pain greatly abated, the patient reported that the swelling and redness were less, and the discharge diminished and free from odor.

8th day. Still improving.

10th day. Increase of pain and discharge.

12th day. Better again.

21st day. Discharge colorless ; pain less ; complexion and appetite a little better ; flesh and strength not changed.

25th day. Decidedly better in strength, appetite, and freedom from pain. Discharge nearly stopped.

27th day. Continues very well. The finger introduced into the rectum finds it still tender, and I was not sure that there was any change in the tumor perceptible to the touch, in the interior of the rectum, but the parts external to the anus were less swollen and less tender.

29th day. Discharge and pain increased.

34th day. About the same as on the twenty-ninth.

From these various facts it is plain that the question of the value of cundurango in cancer is not yet decided.

Some patients have, during its use, experienced a remarkable improvement, while others have felt no change whatever, moreover there has not been time since its introduction to determine whether any case is finally cured : our only course therefore is to continue our experiments, until it is proved to be either a valuable remedy or else an exploded humbug.

No. 6 Sixteenth St., Chicago.

